# Obesity, inflammation, and depression in adolescents

**DOI:** 10.3389/fpsyt.2023.1221709

**Published:** 2023-09-28

**Authors:** Caleb McLachlan, Richard Shelton, Li Li

**Affiliations:** Department of Psychiatry and Behavioral Neurobiology, University of Alabama at Birmingham, Birmingham, AL, United States

**Keywords:** adolescents, depression, obesity, inflammation, association

## Abstract

**Background:**

The prevalence of depression and obesity among adolescents has markedly increased over the last few decades. A bidirectional relationship has been proposed between depression and obesity in adolescence, but it remains poorly understood. Inflammation is a phenomenon that has been implicated in both disorders. Thus, a cross-sectional study was designed to investigate inflammation as a factor in the association between obesity and depression. The goal of this study is to better understand the interplay between these two disorders.

**Methods:**

The study sample consisted of female and male, black and white adolescents aged 15–18 years. Participants were diagnosed with major depressive disorder (MDD) according to the Diagnostic and Statistical Manual of Mental Disorders-5. Depression severity was determined using the Quick Inventory of Depressive Symptomatology (QIDS). Participants completed the Childhood Trust Events Survey (CTES) and received an Early Life Stress (ELS) score based on the survey results. Those with a score of ≥4 were placed in the ELS group and those with a score ≤ 3 were placed in the non-ELS group. Anthropometric measures and a Dual Energy X-ray Absorptiometry (DEXA) scan were performed for body composition. Blood samples were collected to measure inflammatory factors.

**Results:**

Adolescents with MDD (*n* = 47) had significantly elevated body mass index (BMI) percentiles compared to the controls (*n* = 47) (77.11 ± 3.58 vs. 59.63 ± 4.40), and increased adiposity measures, including total fat (*p* = 0.016), trunk fat (*p* = 0.016), and trunk/total fat ratio (*p* = 0.021). Levels of C-reactive protein, tumor necrosis factor-alpha, interleukin-6, leptin, and adiponectin varied significantly between the MDD and control groups, however, significance was not retained when BMI percentile and ELS score were controlled. There was a significant and positive relationship between QIDS and multiple measures of adiposity such as BMI percentile, visceral abdominal tissue, and trunk/total ratio. Depression severity was best predicted by ELS score, visceral adipose tissue, and adiponectin level.

**Conclusion:**

Adolescents with MDD had increased levels of inflammatory factors and many measures of adiposity. Thus, the treatment of adolescent depression should include a focus on managing body composition and reducing chronic inflammation to potentially improve treatment outcomes.

## Introduction

Depression is a major public health concern and also a major source of global health burden, negatively impacting many aspects of daily life (1). The prevalence of depression has increased in both adults and adolescents (2). Data from the National Surveys on Drug Use and Health in the United States showed an increase from 9% in 2004 (2.2 million people) to 15.7% in 2019 (3.8 million people) in the 12-month prevalence of major depressive episodes in adolescents (defined as 12–17 years) (2). As the rate of adolescent depression continues to rise, so do its associated burdens. Adolescent depression negatively impacts both physical health and daily function (3, 4). Additionally, adolescent depression is frequently comorbid with other chronic health conditions, including diabetes, juvenile idiopathic arthritis, and obesity (5, 6).

Like depression, the prevalence of obesity is also rising among adolescents in the United States. Obesity in adolescence has more than quadrupled since 1966 and recent data from The National Health and Nutrition Examination Survey (NHANES) in 2015–2016 showed a 22.2% prevalence of obesity in adolescence (defined as 12–19 years) (7, 8). Obesity in adolescence increases morbidity and mortality in many ways, impacting all major organ systems (9). Prior research in adults has clearly shown that obesity is associated with depression (10, 11). Similarly, studies have demonstrated a close relationship between these disorders in adolescents (12). However, the literature and understanding of why this relationship occurs is much less defined.

Prior research has indicated that inflammation may link depression and obesity (13). Several lines of evidence even suggest that systemic inflammation may be a causal factor for depression in some people (14). For example, medical illnesses that cause increases in inflammatory factors such as interleukin-6 (IL-6), tumor necrosis factor-α (TNF-α), and C-reactive protein (CRP) increase the risk for depression (15). Furthermore, a meta-analysis of 22 studies concluded that depression in children and adolescents is associated with increased inflammatory factors such as IL-6 and CRP (16). The study also demonstrated a bidirectional longitudinal relationship between depression and inflammation in youth (16), which has also been shown in adults (17). Similarly, studies have demonstrated an association between obesity and inflammation (18, 19). Obesity leads to immune cell dysfunction which results in changes in inflammatory factors and acute phase reactant levels. For example, adipocytes secrete pro-inflammatory cytokines, including IL-6, TNF-α, IL-1β, and CRP (20, 21). Additionally, adipose tissue synthesizes its own unique pro-inflammatory molecules in the form of adipokines (22). The studies demonstrate that inflammation is a phenomenon shared by both obesity and depression.

Because it has already been established that obesity and depression are disorders that induce inflammatory states within the body, inflammation could be a mediator of the bidirectional relationship between the two disorders. Preclinical studies show that obesity is associated with increased risk for depression (23). However, clinical studies have been contradictory when trying to identify mediators of this relationship. For example, some studies have shown an association between depression and inflammation while others have found no association (24). To further confound researchers, it has been demonstrated that reducing inflammation alleviates depressive symptoms (25). The aim of the current study is to further explore the relationship between depression, obesity, and inflammation in adolescents since it is understudied. This study intended to address the relationship between depression, obesity, and inflammation in adolescents.

## Methods

### Participants

This was a cross-sectional study design. The study was approved by the Institutional Review Board (#161028005) at the University of Alabama at Birmingham. Participant recruitment and subsequent data collection occurred prior to the COVID-19 pandemic. Adolescents were recruited in Birmingham, Alabama, via advertisement flyers. Written informed consent was provided by both legal guardians and adolescent participants after the study was described by study staff and questions were answered. Participants were either Black or white, male or female, and their ages ranged from 15 to 18 years old. All participants were physically healthy or had stable medical conditions. Exclusion criteria included the following: a history of bipolar disorder, psychotic disorders, primary anxiety disorders, or substance use disorders; current suicidal ideation; a history of endocrine, cardiovascular, or inflammatory disease; having taken antibiotics within 21 days of the study; and females who were pregnant or nursing. Of 105 consented participants, five participants were excluded for not completing the study, five for substance use, and one for a diagnosis of bipolar disorder. Thus, 94 participants completed the study for data analysis. Participating adolescents were also interviewed by a board-certified psychiatrist who evaluated and diagnosed any psychiatric disorders, including MDD, using the Diagnostic and Statistical Manual of Mental Disorders-5 (26). They were then divided into two groups: participants with a diagnosis of MDD (MDD group, *n* = 47) and without a diagnosis of MDD (control group, *n* = 47).

### Assessments

Depression severity was measured in participants using the Quick Inventory of Depressive Symptomatology-Self Report (QIDS-SR), a tool that has been validated for use in adolescents (27). The Childhood Trust Events Survey (CTES) is a self-report screening survey to assess a child’s exposure to traumatic events and has been recommended for use in adolescents (28). It includes 7 categories of adverse events, including physical abuse, emotional abuse, sexual abuse, alcohol exposures, family member in prison, ill caregivers, and loss/separation from caregivers. Each category receives a score of 1 or 0. If any question in the category is answered yes, then the score for that category will be 1. If all questions in the category are answered no, then the score for that category will be 0. All the numbers in the score column are added up to calculate the total score. Total scores range from 0 to 7, with higher scores reflecting less trust and increased Early Life Stress (ELS). ELS status is determined by total score in which participants with a score ≥ 4 were considered as ELS status and those with a score < 4 were considered as non-ELS status.

### Anthropometrics and dual-energy X-ray absorptiometry scan

Measures for height, weight, and waist/hip circumferences were taken in duplicate and averaged for each participant. Height was measured to the nearest 0.1 cm by using a mounted stadiometer. Weight was determined to the nearest 0.1 kg by using an electronic scale. Ten percent of height, weight, and waist circumference measurements were repeated by a second research staff member to assess inter-rater reliability. Body mass index (BMI) was calculated as weight divided by height squared (m^2^) (Liu et al.), and BMI percentile was determined using the Center for Disease Control and Prevention age-based growth charts (29).

Participants also underwent a Dual-energy X-ray absorptiometry (DEXA; Lunar iDXA, GE-Healthcare Madison, WI) scan with a total body scanner to measure body composition. Participants were required to wear light clothes, remove all metal objects from their body, and lie supine with arms at their sides while undergoing the iDXA scan. iDXA allows the simultaneous measurement of bone mass, fat mass, and lean body mass from the ratio of attenuation of two energy beams passing through the body (30). CoreScan software was used to estimate the visceral adipose amount based on measurement of abdominal area and subcutaneous adipose tissue.

### Blood draw and measurements

Fifteen milliliters of blood were drawn from each participant. Blood samples were centrifuged at 3,000 g for 15 min. After centrifugation, the samples were divided into two aliquots and frozen at −80°C until analysis. The following inflammatory factors were measured and analyzed using a Meso Scale discovery multiplex spot assay and MSD Discovery Workbench software (Gathersburg, MD): IL-6, IL-8, IL-10, interferon-gamma, and TNF-α. Their concentrations were expressed in pg./ml. Plasma concentrations of leptin (ng/mL) and adiponectin (μg/mL) were assayed using commercially available radioimmunoassay kits according to the procedures supplied by the manufacturer (Millipore Corp, Billerica, MA). CRP (mg/L) was analyzed using immunoassay on a Stanbio Sirrus Analyzer (Stanbio Laboratory, Boerne, TX). All samples were measured twice, and the mean of both samples was reported.

### Statistical analysis

The Statistical Package for the Social Sciences (SPSS, v26) was used to conduct all analyses and significance was set at a *value of p* ≤ 0.05. Kolmogorov-Smirnoff tests were used to test all variables for normal distribution. If a variable did not conform to normality, it was log-transformed for distribution normality, including the inflammatory factors. Categorical variables including gender, race, ELS status, employment status, smoking status, education in legal guardians, and use of antidepressants were compared between the two groups using a Chi-square test. An independent *t*-test was used for continuous data, including age, family income, and QIDS scores. An Analysis of covariance (ANCOVA) was used to compare differences in anthropometric measures, DEXA measures, and inflammatory factors between the MDD and control groups while controlling for race, ELS score, and anti-depressant use. Additionally, ANCOVA was used while controlling for BMI percentile when inflammatory factors were compared between the two groups. A Pearson correlation analysis was used to assess the association between QIDS score and multiple measures of adiposity. Stepwise multiple linear regression analysis was used to identify the independent variables that best predicted depression severity in the MDD group. Variables that may contribute to depression were entered into the model, including all measured inflammatory factors, age, race, gender, anti-depressants, body composition measures, and ELS scores. Regression analyses and the Sobel test were used to explore the mediating effect of inflammation on the relationship between obesity and MDD.

## Results

### Participants’ characteristics

A summary of the 94 participants is presented in Table 1. There were no significant differences in gender, age, current smoking status, family income, guardian education and employment status between the two groups. Compared with the control group, there were more white participants in the MDD group. More participants in the MDD group reported to have a history of ELS and were taking antidepressants.

**Table 1:** Participant characteristics.

	MDD (*n* = 47)	CTL (*n =* 47)	*p-*value
Male/female (*n*)	19/28	22/25	0.53
White/black (*n*)	36/11	15/32	**<0.001**
Early life stress (*n*)	26	4	**<0.001**
Current smoker (*n*)	3	2	0.65
Family income ($)	62,742 ± 7,448	59,053 ± 8,200	0.54
Guardian education ≥12 years (*n*)	34	38	0.42
Guardian unemployed (*n*)	12	14	0.74
Age (years)	16.0 ± 0.1	16.0 ± 0.1	1.00
Current anti-depressant use (*n*)	33	2	**<0.001**

The *p*-value comes from Chi-squared test for the categorical variables and *t*-test for continuous variables. Data is presented as mean ± standard deviation or number. MDD, major depressive disorder; CTL, control. Bolded values indicate those that are statistically significant (*p* value < 0.05).

### Relationship between depression and body composition

Anthropometric and DEXA scan data of the participants is shown in Table 2. After controlling for race, ELS score, and antidepressant use, several adiposity measures were significantly greater in the MDD group, including but not limited to BMI percentile (*p* = 0.005), total fat (*p* = 0.016), and trunk/total fat ratio (*p* = 0.021). Compared with the control group, VAT mass was greater, but not significantly, in the MDD group (*p* = 0.057).

**Table 2:** Descriptive statistics of body composition measures.

	MDD	CTL	*p-*value
BMI percentile	77.11 ± 3.58	59.63 ± 4.40	**0.005**
Waist circumference (inch)	36.24 ± 1.54	30.40 ± 0.73	**0.006**
Waist/hip ratio	0.87 ± 0.02	0.84 ± 0.01	0.11
VAT mass (g)	532 ± 82.33	254.83 ± 39.73	0.057
Total fat (kg)	28.8 ± 2.4	19.0 ± 1.7	**0.016**
Trunk fat (kg)	14.1 ± 1.4	8.3 ± 5.9	**0.016**
Leg fat (kg)	10.7 ± 0.8	7.7 ± 0.6	**0.025**
Trunk/total fat ratio	0.46 ± 0.01	0.41 ± 0.01	**0.021**
Total lean mass (kg)	47.1 ± 1.4	43.2 ± 1.8	0.20
Extremity lean mass (kg)	22.3 ± 0.8	21.2 ± 0.8	0.17
Gynoid fat (g)	5030.78 ± 401.07	3,349.15 ± 301.85	**0.05**
Android fat (g)	2248.72 ± 254.85	1217.09 ± 164.26	**0.027**

The *p*-value comes from an ANCOVA controlling for race, early life stress score, and antidepressant use. Data is presented as mean ± standard deviation. MDD, major depressive disorder; CTL, control; BMI, body mass index; VAT, visceral adipose tissue; g, grams; kg, kilograms. Bolded values indicate those that are statistically significant (*p* value < 0.05).

**Figure 1:**
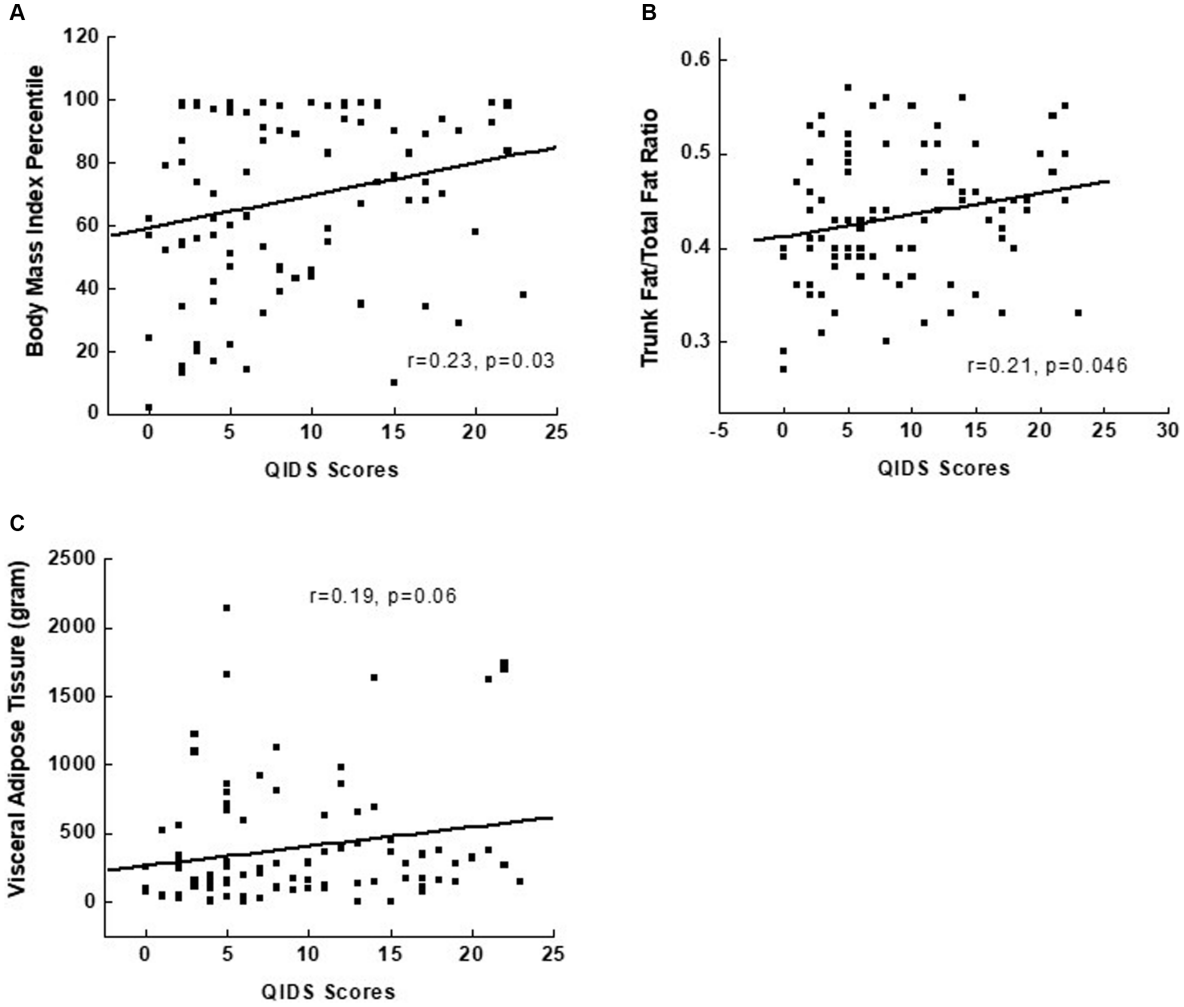
Relationships between the severity of depression, measured by the Quick Inventory of Depressive Symptomatology, and measures of body composition in depressed adolescents, including body mass index percentile in **(A)**, trunk/total fat ratio in **(B)**, and visceral adipose tissue in **(C)**.

Pearson correlation was conducted between depression severity measured by the QIDS score and multiple measures of adiposity. Among all measures, BMI percentile, VAT mass, trunk/total ratio, and VAT had a significant and positive correlation, albeit weak, with QIDS score (Figure 1).

### Relationship between depression and inflammation

Data for inflammatory factors of the participants is shown in Table 3. A *t*-test between the two groups showed significantly elevated levels of TNF-α (*p* = 0.007) and leptin (*p* = 0.024) in the MDD group in contrast to the control group. Significance was not retained for these inflammatory factors after controlling for race, anti-depressant use, and ELS score. Another ANCOVA was run in which BMI percentile was controlled, TNF-α level was significantly greater in the MDD group (*p* = 0.014).

**Table 3:** Descriptive statistics of the inflammatory factors.

	MDD	CTL	*p*1 value^*^	*p*2 value^*^	*p*3 value^*^
CRP	3.52 ± 0.92	1.92 ± 0.47	0.24	0.34	0.98
IFN-γ	5.01 ± 0.79	3.33 ± 0.46	0.06	0.81	0.13
TNF-α	2.35 ± 0.11	2.04 ± 0.25	**0.007**	0.090	**0.014**
IL-6	0.86 ± 0.11	0.77 ± 0.11	0.54	0.081	0.46
IL-8	8.24 ± 0.53	10.14 ± 1.57	0.47	0.87	0.55
IL-10	0.44 ± 0.06	0.39 ± 0.06	0.41	0.47	0.35
Leptin	52.15 ± 7.24	35.17 ± 6.70	**0.024**	0.24	0.37
Adiponectin	10.89 ± 0.76	13.27 ± 1.26	0.23	0.19	0.99

Data is presented as mean ± standard deviation. MDD, major depressive disorder; CTL, control; CRP, C-reactive protein; IFN-γ; interferon-gamma; TNF-α, tumor necrosis factor-alpha; IL-6, interleukin-6; IL-8, interleukin-8; IL-10, interleukin-10.^*^p1 comes from a *T*-test.^*^p2 comes from an ANCOVA controlling for race, anti-depressant use, and early life stress score.^*^p3 comes from an ANCOVA controlling for body mass index percentile. Bolded values indicate those that are statistically significant (*p* value < 0.05).

Stepwise multiple linear regression analysis in the MDD group indicated that depression severity, i.e., QIDS score, was best predicted by ELS score, VAT, and adiponectin level (Table 4). Mediation analysis showed that adiposity, including BMI percentile, VAT, and trunk/total fat ratio, had some indirect effects (0.0006, 0.12, and 4.18, respectively) on QIDS score, but these effects were not significant.

**Table 4:** Stepwise linear regression analysis predicting depression severity.

	MDD sample (*n* = 47)			
Dependent variable	Significant predictors	Unstandardized β	*R^2^*	95% confidence interval for β
QIDS	ELS score	4.9	0.23	2.97–6.79
QIDS	ELS score, VAT	7.6	0.34	5.72–10.05
QIDS	ELS score, VAT, adiponectin	10.1	0.40	7.54–13.33

MDD, major depressive disorder; QIDS, Quick Inventory of Depressive Symptomatology; ELS, early life stress; VAT, visceral adipose tissue.

## Discussion

In this cross-sectional study, the relationship between obesity, inflammation, and depression in adolescents was studied. A variety of measures for body composition, including BMI percentile, total fat, and trunk/total fat ratio, were found to be greater in adolescents with depression compared with their non-depressed counterparts. Some measured inflammatory factors were also elevated in adolescents with MDD compared with controls. Furthermore, depression severity measured with QIDS was positively correlated with BMI percentile, VAT, trunk/total fat ratio. Additionally, depression severity was best predicted by ELS scores, VAT, and adiponectin level. Consistent with previous studies, this study demonstrated a significantly greater amount of adiposity in the MDD group (5, 31).

Furthermore, our study also found significant associations of adolescent depression with other measures of adiposity that were not previously studied, such as the gynoid region which includes the hips and upper thighs and the android region. Additionally, depression severity showed a significantly positive correlation with BMI percentile and VAT. Thus, our study was able to confirm previous findings while also adding further findings to the current understanding of increased adiposity in participants with depression. Future studies may need to focus on the exact role of each measured body composition in adolescent depression and potential therapeutic impact.

The results of our study support the idea that adolescent depression is associated with harmful phenotypic changes in body composition, including VAT. For example, it is well established that the increased VAT in adults is associated with a plethora of adverse outcomes such as insulin resistance, type 2 diabetes, atherogenic dyslipidemia, cardiovascular disease, and even cancer (32–35). Similarly, VAT has also been demonstrated to be a more accurate predictor of insulin resistance than total fat in the child and adolescent population, with VAT becoming a more important marker for metabolic dysfunction as a child progresses from pre-pubescent to post-pubescent (36). Our findings indicate that VAT is associated with the severity of depression in adolescents and VAT is a predictor for depression as well. Combined results highlight the unique role of VAT compared with other adiposity measures, and thus highlight the importance of assessing the regional changes in adiposity rather than holistic changes in metabolic syndrome and depression management.

Evidence has shown that depression in adults is associated with elevated inflammatory markers (19, 37–39). However, the evidence for a relationship between depression in adolescents and elevated inflammatory markers has been inconsistent. For example, some studies have shown immune dysregulation and subsequent elevated CRP to be associated with adolescent depression, while others have found no associations between adolescent depression and inflammatory markers (40, 41). A meta-analysis examining the relationship between depression and inflammation in children and adolescents found that depression was significantly associated with elevated levels of CRP and IL-6. Furthermore, this study reported that across the included studies, TNF-α level fell just short of having a significant association with adolescent depression (16). We compared all measured inflammatory factors between adolescents with- and without-MDD. Among them, leptin, TNF-α, and IFN-γ were different, however, only a marginally significant difference remained for TNF-α after adjusting for covariates. In contrast to prior findings, our study found no significant elevation of adiponectin and IL-6 in adolescents with MDD. There are several reasons that may contribute to heterogeneous findings on adolescent depression and inflammation. One reason could be due to the relatively small sample size in our current study. Another reason could be due to differences in methodology when data is collected and analyzed. For example, these studies differ from our study in that they did not control BMI percentile and ELS score when comparing inflammatory factors between the groups although both are related with depression and inflammation (40, 41). This demonstrates the need for further investigation to define the relationship between adolescent depression and inflammatory markers.

Consistent with the literature, ELS score was associated with depression and one of factors that could predict depression severity in our study (42–44). Additionally, we found that significant differences in inflammatory factor levels between the control and MDD groups were not retained when ELS was adjusted. This is indicative of the potential role of ELS in inflammation and depression. Indeed, previous studies have shown the association of ELS with chronic inflammation in adults although heterogenous results have been reported in children and adolescents (45–48). Thus, future studies are warranted to further explore the relationship between ELS and inflammation, especially in children and adolescents. These studies should also include different types of ELS while analyzing age-related and sex-related effects on this relationship.

Our study had some limitations that should be noted when interpreting the results. The first is that our sample size is relatively small, and our results may need to be reproduced in a larger clinical trial. Secondly, this was a cross-sectional study design so the cause-and-effect of associations cannot be determined from our results. Finally, QIDS is a measure we used to assess the severity of depressive symptoms. The limitation with this tool is that it is a self-report by the participants. However, it should be noted that the QIDS has been determined to be a reliable and valid assessment of depressive symptomatology in adolescents (27).

In this study, we furthered the investigation of the relationship between adolescent depression, inflammation, and obesity. Our study indicates that the treatment of adolescent depression should focus not only on depression but should also manage body composition to prevent harmful outcomes associated with increased fat mass, especially VAT. Furthermore, our findings provide some insights for potential novel therapeutic approaches to treat adolescent depression, i.e., anti-inflammatory therapy.

## Data availability statement

The raw data supporting the conclusions of this article will be made available by the authors, without undue reservation.

## Ethics statement

The study was approved by the Institutional Review Board (#161028005) at the University of Alabama at Birmingham. The studies were conducted in accordance with the local legislation and institutional requirements. Written informed consent for participation in this study was provided by the participants’ legal guardians/next of kin.

## Author contributions

CM analyzed the data and drafted, edited, and approved the final manuscript. RS conceptualized and designed the study and edited and approved the final manuscript. LL conceptualized and designed the study, collected and analyzed the data, and reviewed, edited, and approved the final manuscript. All authors contributed to the article and approved the submitted version.
